# Genome-Wide Identification and Analysis of Chitinase GH18 Gene Family in *Mycogone perniciosa*

**DOI:** 10.3389/fmicb.2020.596719

**Published:** 2021-01-11

**Authors:** Yang Yang, Frederick Leo Sossah, Zhuang Li, Kevin D. Hyde, Dan Li, Shijun Xiao, Yongping Fu, Xiaohui Yuan, Yu Li

**Affiliations:** ^1^Engineering Research Center of Chinese Ministry of Education for Edible and Medicinal Fungi, Jilin Agricultural University, Changchun, China; ^2^Guizhou Key Laboratory of Edible Fungi Breeding, Guizhou Academy of Agricultural Sciences, Guiyang, China; ^3^College of Plant Protection, Jilin Agricultural University, Changchun, China; ^4^Shandong Provincial Key Laboratory for Biology of Vegetable Diseases and Insect Pests, College of Plant Protection, Shandong Agricultural University, Tai’ an, China; ^5^Center of Excellence in Fungal Research, Mae Fah Luang University, Chiang Rai, Thailand

**Keywords:** *Mycogone perniciosa*, chitinase glycoside hydrolase 18 gene family, phylogeny, transcriptome, expression pattern

## Abstract

*Mycogone perniciosa* causes wet bubble disease in *Agaricus bisporus* and various Agaricomycetes species. In a previous work, we identified 41 GH18 chitinase genes and other pathogenicity-related genes in the genome of *M*. *perniciosa* Hp10. Chitinases are enzymes that degrade chitin, and they have diverse functions in nutrition, morphogenesis, and pathogenesis. However, these important genes in *M*. *perniciosa* have not been fully characterized, and their functions remain unclear. Here, we performed a genome-wide analysis of *M*. *perniciosa* GH18 genes and analyzed the transcriptome profiles and GH18 expression patterns in *M*. *perniciosa* during the time course of infection in *A*. *bisporus*. Phylogenetic analysis of the 41 GH18 genes with those of 15 other species showed that the genes were clustered into three groups and eight subgroups based on their conserved domains. The GH18 genes clustered in the same group shared different gene structures but had the same protein motifs. All GH18 genes were localized in different organelles, were unevenly distributed on 11 contigs, and had orthologs in the other 13 species. Twelve duplication events were identified, and these had undergone both positive and purifying selection. The transcriptome analyses revealed that numerous genes, including transporters, cell wall degrading enzymes (CWDEs), cytochrome P450, pathogenicity-related genes, secondary metabolites, and transcription factors, were significantly upregulated at different stages of *M*. *perniciosa* Hp10 infection of *A*. *bisporus*. Twenty-three out of the 41 GH18 genes were differentially expressed. The expression patterns of the 23 GH18 genes were different and were significantly expressed from 3 days post-inoculation of *M*. *perniciosa* Hp10 in *A*. *bisporus*. Five differentially expressed GH18 genes were selected for RT-PCR and gene cloning to verify RNA-seq data accuracy. The results showed that those genes were successively expressed in different infection stages, consistent with the previous sequencing results. Our study provides a comprehensive analysis of pathogenicity-related and GH18 chitinase genes’ influence on *M*. *perniciosa* mycoparasitism of *A. bisporus*. Our findings may serve as a basis for further studies of *M*. *perniciosa* mycoparasitism, and the results have potential value for improving resistance in *A*. *bisporus* and developing efficient disease-management strategies to mitigate wet bubble disease.

## Introduction

The button mushroom (*Agaricus bisporus*) is one of the most widely cultivated and consumed edible mushrooms in the world. Production of button mushrooms in China has rapidly increased in recent years as a result of the expanded area of cultivation and the adoption of technology for improved commercial cultivation (He et al., 2014; Sonnenberg et al., 2017; Li et al., 2018; McGee, 2018). However, diseases caused by fungi, bacteria, and viruses are major constraints to *A*. *bisporus* production worldwide, often leading to serious economic losses (Fletcher et al., 1989; Largeteau and Savoie, 2010; Kouser et al., 2013). Wet bubble disease (WBD) is one of the most devastating diseases of *A*. *bisporu*s, causing yield losses of 15–30% under favorable conditions and up to 75% or total crop loss in the most severe cases (Zhou et al., 2015). WBD is characterized by wet bubbles, malformation, white, fluffy mycelial growth, copious amber droplets (diseased carpophores exuding a brown malodorous liquid), and flocculent mycelia on most substrates (Fletcher et al., 1995; Sharma and Kumar, 2000; Umar et al., 2000). *Mycogone perniciosa* (teleomorph: *Hypomyces perniciosus*) is the causal agent of WBD, and it infects a variety of mushrooms (Zhang et al., 2017a,b; Carrasco et al., 2019). *M*. *perniciosa* is a fungicolous fungus belonging to the order Hypocreales (Ascomycota) in the family Hypocreaceae.

*M. perniciosa* is mainly controlled by cultural practices and the application of fungicides (Brankica et al., 2009). Varying levels of WBD resistance have been identified in the *A*. *bisporus* germplasm collection in China; however, no major resistance gene has been identified (Fu et al., 2016). The genome of *M*. *perniciosa* contains many genes implicated in pathogenicity (Li et al., 2019), but the regulation of these genes in the pathogenesis toward *A*. *bisporus* is still unclear. In addition, comparative genomics analysis of *M*. *perniciosa* has revealed gene expansion and positive selection of many genes, including GH18 chitinase, peptidase, and secondary metabolite genes (Li et al., 2019). Gene expansion and positive selection contribute to the evolution of virulence genes in microbial pathogens and to the adaptation to different environmental niches through the infection process and via escape from the host defense response (Yoshizaki et al., 2019).

Chitinase (EC 3.2.1.14) is a glycosyl hydrolase enzyme that degrades chitin (Hamid et al., 2013). Chitin is the main structural component of fungal cell walls and the exoskeleton of animals, including worms and arthropods (Mauch et al., 1988). Chitinases are found in various organisms, including those that do not contain chitin (Rathore and Gupta, 2015). Chitinases of pathogenic fungi not only play vital roles in spore germination, septum formation, cell division, and morphogenesis, but the enzymes are also important in the host interaction (Elad et al., 1982, 1983; Inbar and Chet, 1995; Chen, 2002; Adams, 2004). In addition to degradation of the host fungal cell wall, chitinases also inhibit hyphae growth and bud tube elongation (Gozia et al., 1993; Stressmann et al., 2004).

Chitinases are classified into two families, namely glycosyl hydrolases 18 and 19, based on amino acid sequence similarity (Henrissat and Bairoch, 1993). The GH18 chitinase gene family is widely distributed in bacteria, fungi, viruses, animals, and higher plants (Henrissat and Bairoch, 1993; Kawase et al., 2004; Seidl, 2008; Hartl et al., 2012; Adrangi and Faramarzi, 2013). Omics and bioinformatics analyses have demonstrated that most fungal chitinases have similar domains that generally contain a signal peptide sequence, a chitinase catalytic domain, a chitin-binding domain, and a short C-terminal domain (van Aalten et al., 2001). Among fungal pathogens, GH18 chitinase gene families are well characterized in *Trichoderma* species, *Fusarium* species, and *Magnaporthe* species (Häkkinen et al., 2012; Han et al., 2019). Currently, 30 chitinase genes have been reported within eight species of *Trichoderma*, including *T. harzianum* (30 genes), *T. virens* (29 genes), and *T. atroviride* (24 genes) (Kubicek et al., 2011, 2019).

The advent of next-generation sequencing technologies has increased the scalability, speed, and resolution of genomic sequencing and reduced genome sequencing cost (Faino et al., 2015). This has rapidly increased fungal genome availability for comparative genomics and genome-wide identification of gene families (Bartholomew et al., 2019). However, few studies have comprehensively examined the structure or the expression of GH18 chitinases of fungal pathogens infecting mushrooms.

In a previous work, we identified 41 GH18 chitinase genes in the genome of *M. perniciosa* (Li et al., 2019). However, the genome-wide identification, functional characterization, and expression of these GH18 chitinase genes were not considered. In this study, we present the first detailed and comprehensive analysis of the GH18 gene family in the genome of *M. perniciosa*. The analyses include chromosome location, phylogenetic analysis, protein structure, and motif composition. Furthermore, we performed a transcriptome analysis of the different infection stages of *M. perniciosa* on *A*. *bisporus* to identify pathogenicity-related differential gene expression and expression patterns of the GH18 genes. In addition, we selected five GH18 genes that represented different types and identified their expression levels by RT-PCR and gene cloning to verify the sequencing. The results can provide important information for identifying essential genes as potential antifungal targets in *M. perniciosa*. Further, the dataset generated in this study may provide a basis for identifying candidate resistant genes in *A. bisporus* against *M. perniciosa* and lay a foundation for further research to improve the resistance of *A. bisporus* to *M. perniciosa*.

## Materials and Methods

### Strains and Culture Conditions

The fungal strains [*A*. *bisporus* strain A3 (CCMJ1009) and *M*. *perniciosa* Hp10] were obtained from the Engineering Research Center of Edible and Medicinal Fungi, Ministry of Education, Jilin Agricultural University (Changchun, Jilin, China). The *M*. *perniciosa* Hp10 used in this study is a highly pathogenic strain (≥90%) able to cause severe disease on all *A*. *bisporus* evaluated to date (Li et al., 2019). All the fungal strains were maintained on potato dextrose agar (PDA) at 25°C. *Escherichia coli* DH5 α and GV pxt19-t vector were purchased from Beijing TransGen Biotech Co., Ltd., and Beijing Dingguo Changsheng Biotechnology Co., Ltd., respectively.

### Mushroom Cultivation, Inoculum Preparation, and Disease Evaluation

Cultivation of *A*. *bisporus* and evaluation of the WBD infection process were conducted at the Mushroom Base of Jilin Agricultural University, Changchun, China, using methods described by Li et al. (2019). *A*. *bisporus* mycelia were inoculated on autoclaved wheat grains to produce spawn. The spawn was inoculated on a (45 × 33 × 25 cm) basket filled with 7.5 kg compost. After the mycelia overgrew the compost, 4 cm thick casing soil was applied to cover the compost. To induce fruiting, the room temperature, relative humidity, and carbon dioxide (CO_2_) concentration were set at 15–18°C, 80–95%, and 1,200–1,500 ppm, respectively.

Spore suspensions of *M*. *perniciosa* Hp10 inoculum were from 7-day-old pure PDA cultures grown at 25°C. The cultures were suspended in 5 ml sterile distilled water (SDW), gently scraped with a glass stick, and filtered through two cheesecloth layers. The spore concentration was determined and adjusted to 1 × 10^5^ spores/ml using a hemocytometer.

When the primordial caps reached 0.5 cm diameter after emergence from the casing soil, approximately 50 ml of *M*. *perniciosa* Hp10 spore suspension was sprayed on the surface of the caps in each basket. Similarly, 50 ml of SDW was sprayed on the surface of the primordial caps in each basket as a negative control. After inoculation, the mushrooms were observed for changes in disease symptoms every 24 h for 20 days by randomly selecting infected fruiting bodies and observing them under a light microscope (Zhang et al., 2017a). Tissues from the important time-points (0-, 3-, 4-, 5-, 10-day old tissues) during *M*. *perniciosa* Hp10 infection on *A. bisporus* were collected, after which the samples were immediately frozen in liquid nitrogen and stored at −80°C until further use. The test was repeated twice, with four baskets for each test. The disease assessment was recorded for only the first flush. After disease development, the pathogen was reisolated, as previously described.

### Identification and Characterization of GH18 Gene Family Members of *M. perniciosa* Hp10

The annotated protein sequences of *M. perniciosa* Hp10 were used as queries for a hidden Markov model (HMM) search against the SwissProt^1^ (Boutet et al., 2016), InterPro^2^ (Mitchell et al., 2019), and carbohydrate-active enzymes databases (CAZy) (Lombard et al., 2013) using HMMER 3.3^3^. The retrieved sequences were searched against the SMART^4^ (Letunic and Bork, 2018) and the National Center for Biotechnology Information (NCBI) Conserved Domain Search Service tool^5^ (Lu et al., 2020) to confirm the conserved domains for the GH18 gene family. The number of amino acids, theoretical molecular weight (MW), and isoelectric point (PI) of the GH18 proteins were predicted using ProtParam^6^ (He et al., 2019). The subcellular localization was predicted using BUSCA^7^ (Savojardo et al., 2018) web server.

### Phylogenetic Analysis

The chitinase GH18 sequences of the longest catalytic conserved domains (>120 amino acids) of *M*. *perniciosa* Hp10 and 15 other fungal pathogens (*Aspergillus fumigatus*, *Beauveria bassiana*, *Cordyceps militaris*, *Fusarium graminearum*, *Fusarium vanettenii*, *Fusarium oxysporum*, *Hirsutella thompsonii*, *Metarhizium robertsii*, *Monosporascus* sp., *Pyricularia oryzae*, *Trichoderma virens*, *Trichoderma parareesei*, *Trichoderma atroviride*, *Neurospora crassa*, and *Trichoderma reesei*) were obtained and used to construct the GH18 phylogenetic tree. The sequences were aligned using Cluster X 2.1 (Larkin et al., 2007), and the phylogenetic trees were constructed based on the alignment using the Neighbor-Joining method (1,000 repeats) with the parameters of the Jones-Taylor-Thornton model, uniform rates among sites, and partial deletion of gaps in MEGA X version 10.1 (Kumar et al., 2018).

### Gene Structure, Conserved Motif Analyses, and Chromosomal Location

The exon and intron structures were identified by aligning the coding sequence of each gene against the genome sequence using the Gene Structure Display Server^8^. The conserved motifs of the genes were predicted using MEME 5.1.1^9^ (Bailey et al., 2006) with default parameters. The secondary structure and tertiary structure of the chitinase GH18 gene family were predicted using Predict Protein software^10^ (Rost et al., 2004) and SWISS-MODEL software^11^ (Biasini et al., 2014), respectively. The MapChart 2.32 software (Voorrips, 2002) was used to visualize the chromosomal distributions of *M. perniciosa* Hp10 GH18 genes based on the gene starting positions and chromosomal lengths.

### Identification of Orthologs and Gene Duplication

OrthoFinder 2 (Emms and Kelly, 2019) was used to determine the orthologous genes and duplicated gene pairs between *M*. *perniciosa* Hp10 chitinase GH18 and the other 15 species. GH18 protein sequences of *M*. *perniciosa* Hp10 were used in reciprocal BLASTP searches with an E value cutoff of 10e-5 and coverage of ≥80% to give lists of BLAST hits and query/target midpoint positions for each chromosome. Genes on the same chromosome separated by two or more genes in a 100 kb region on a chromosome (Nei and Gojobori, 1986) were considered as tandem array genes. The ratios of non-synonymous to synonymous nucleotide substitution rates (Ka/Ks) of the duplicated genes in *M*. *perniciosa* Hp10 were calculated using Ka/Ks Calculator 2.0 (Liberles, 2001; Siltberg and Liberles, 2002).

### Expression Pattern Analysis of the GH18 Gene Family of *M. perniciosa* Hp10

Total RNA was extracted from 100 mg of *A*. *bisporus* tissues at each time-point (0-, 3-, 4-, 5-, and 10-day old tissues) using TRIzol reagent (Invitrogen, Carlsbad, CA, United States) following the manufacturer’s instructions. For each time point, nine tissues were randomly chosen; RNA was extracted, and then equal amounts of RNA from nine tubes were mixed into three new tubes. The purity, concentration, and integrity of RNA samples were determined using a NanoDrop ND-1000 spectrophotometer (NanoDrop Technologies, Wilmington, DE, United States) and an Agilent Bioanalyzer 2100 system (Agilent Technologies, CA, United States). The first-strand cDNA was generated using transcript one-step gDNA removal and cDNA synthesis SuperMix kit (TransGen Biotech Co., Ltd., Beijing, China) following the manufacturer’s instructions. cDNA libraries were constructed using a NEBNext^®^Ultra^TM^ RNA Library Prep Kit for Illumina^®^ (NEB, United States) following the manufacturer’s recommendations. The cDNA libraries were sequenced on an Illumina HiSeq X-ten platform with 150 bp paired-end reads at the Novogene Biotech Company (Beijing, China).

Two biological repeats were established for each treatment. After sequencing, low-quality reads and adapter sequences were removed from the raw data using the NGS QC Toolkit^12^ (Patel and Jain, 2012). The clean data were then mapped to the *M. perniciosa* Hp10 and *A. bisporus* H97 genome sequences using TopHat^13^ (Trapnell et al., 2009). The differentially expressed genes (DEGs) analysis was performed with DESeq software (version 1.18.0) (Anders and Huber, 2010). The Fragments Per Kilobase of transcript per Million mapped reads (FPKM) method (Trapnell et al., 2010) was used to obtain the expression levels and to calculate the differential expression multiples among different samples. The false discovery rate (FDR) was used to test the multiple hypotheses of the calculated results, FDR not greater than 0.001, and | log2 ratio | ≥ 1 were defined as the threshold to screen differentially expressed genes (DEGs) and were employed to obtain significantly differentially expressed genes among samples (Padj < 0.05).

All of the DEGs were functionally annotated by mapping to the Gene Ontology (GO), Kyoto Encyclopedia of Genes and Genomes (KEGG)^14^, and InterProScan databases (Ashburner et al., 2000; Jones et al., 2014; Kanehisa et al., 2015; The Gene Ontology Consortium, 2018) using BLASTX program with an E-value cutoff of 10^–5^ and identity cutoff of 40%. The Pretty Heatmaps (pheatmap) package (Yao and Liu, 2018) in R software was used to draw the expression pattern of members of the GH18 gene family. These RNA-sequencing data have been submitted to the NCBI SRA database (SRP190007).

### Gene Cloning and Analysis of the GH18 Genes

To validate the RNA-seq data, five differentially expressed GH18 genes at different time points (0, 3, 4, 5, and 10 days) of *M. perniciosa* infection of *A. bisporus* were analyzed by PCR and RT-PCR. Specific primers (Table 1) were designed for the five GH18 genes using Primer Premier 5.0 software (Liu et al., 2015). Approximately 200 ng/μL of DNA and cDNA products were used as templates for the PCR and RT-PCR. The reactions were performed in 25 μL containing 2 μL template DNA, 12.5 μL of Premix Taq (TaKaRa, Da Lian, China), 1 μL (10 μM) of each primer, and 10.5 μL of RNase-free water. The reactions were performed in a Bio-Rad T100 thermal cycler (Bio-Rad Lab. Inc., Ltd., California, United States) with the following conditions: 94°C for 5 min, followed by 30 cycles of 94°C for 30 s and 60°C for 30 s, then 72°C for 30 s, and a final extension at 72°C for 10 min. The amplified products were detected by 1% agarose gel electrophoresis, purified using an Axyprep DNA Gel Extraction Kit (Axygen Scientific, Inc., California, United States), and cloned into a pXT19-T Vector (Beijing Dingguo Changsheng Biotechnology Co., Ltd., Beijing, China) followed by transformation into *E. coli* DH5α. The positive clones were screened by LB liquid medium containing ampicillin and confirmed by PCR amplification and agarose gel electrophoresis. Three positive plasmid samples were sequenced using the five GH18 specific primers at Sangon Biotech Co., Ltd. (Shanghai, China), and the sequences were analyzed using DNAMAN 6.0 (Lyu et al., 2017). Two biological replicates and three technical replicates of each sample were used for both the PCR and RT-PCR reactions.

**TABLE 1:** Oligonucleotide primers for gene cloning.

**Primer**	**Sequence(5′–3′)**	**Number (bp)**
LD01F	ATGCTCGGTTTTCTCACCAAGT	22
LD01R	TTAGTTCAGACCGTTCTTGATGTT	24
LD02F	ATGCGTTCCTCAATGCTC	18
LD02R	TCACGACAGCGATTCAAC	18
LD03F	ATGACACGTCTTCTCGAAG	19
LD03R	TCAGAGCCCGAGCCGC	16
LD04F	ATGGTTCGCTCTTTGGCTTCT	21
LD04R	TTAGTTGAGATAGCCGACA	19
LD05F	ATGAAGTCCCTGTTCCTAT	19
LD05R	CTATGCATTCACCATTGCC	19

## Results

### Genome-Wide Identification and Characterization of GH18 Genes in *M. perniciosa* Hp10

**FIGURE 1:**
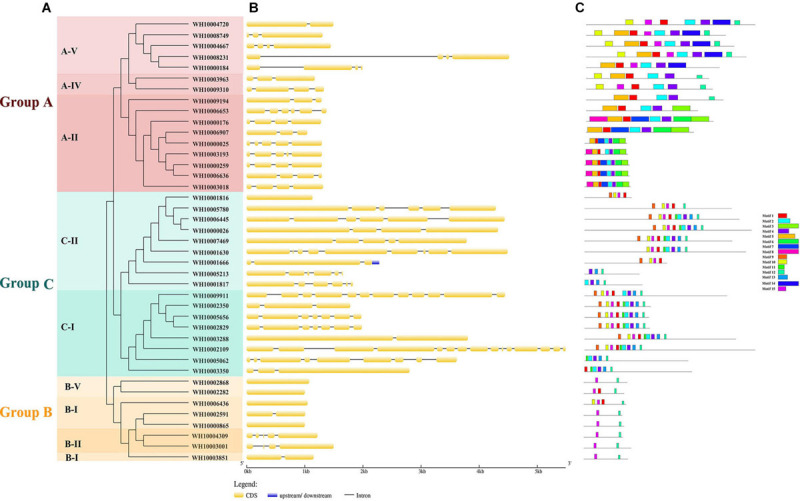
Analysis of 41 GH18 genes. **(A)** Phylogenetic tree. Different colors (Red, green, and orange) indicating different groups and different shades of the same color represent different subgroups. **(B)** Gene structure. CDS denote exons. **(C)** Motif analysis. The length and different colors of boxes denote motif length and different motifs, respectively.

A total of 63 putative GH18 gene sequences were obtained from the genome of *M. perniciosa* Hp10 after an HMM search of CAZy, InterPro, and SwissProt databases. All candidate GH18 sequences were further analyzed using the CDD and SMART databases to confirm the presence of conserved domains. Forty-one putative GH18 genes were obtained after eliminating short length (100 bp) and low identity sequences (Table 2). Based on the conserved domains, the 41 GH18 genes were divided into three groups and eight subgroups (Figure 1A), in which 18 genes contained signal peptides at the N-terminus, and 14 genes had small domain CBM1, ChtBD1, or LysM. Sixteen genes (gene length 1,044–4,577 bp) belonged to group A that contained three subgroups (A-II, A-IV, and A-V); each group contained Glyco_hydro_18. Eight genes (gene length 1,002–1,498 bp) belonged to group B, which contains three subgroups (B-I, B-II, and B-V); each contained GH18_hevamine_XipI_class_III, GH18_CTS3_chitinase, or GH18_chitinase_D-like, and one gene (WH10003001) of subgroup B-II contained a small conserved domain CBM1 at the C-terminus. Seventeen genes (gene length 1,134–5,487 bp) belonged to group C that contained two subgroups (C-I and C-II). Each group contained GH18_zymocin_alpha or GH18_chitolectin_chitotriosidase conserved domain, and some had the ChtBD1 or LysM domain. Five genes of subgroup C-II had two concurrent domains.

**TABLE 2:** Gene sequence analysis of 41 GH18 genes of *M. perniciosa* Hp10.

**Genes**	**Group**	**Location**			**Gene(bp)**	**CDS (bp)**	**Introns number**	**Conserved domains**
		**Utg**	**Start**	**End**				
WH10000025	A-II	327	10,006	11,300	1,295	1,029	4	Glyco_hydro_18
WH10000176	A-II	195	6,67,605	6,68,886	1,282	1,086	3	Glyco_hydro_18
WH10000259	A-II	195	11,50,338	11,51,632	1,295	1,089	3	Glyco_hydro_18
WH10003018	A-II	19	34,57,821	34,59,136	1,316	1,113	3	Glyco_hydro_18
WH10003193	A-II	44	37,073	38,367	1,295	1,044	4	Glyco_hydro_18
WH10006636	A-II	81	40,45,049	40,46,343	1,295	1,089	3	Glyco_hydro_18
WH10006653	A-II	81	43,88,847	43,90,221	1,375	954	5	Glyco_hydro_18
WH10006907	A-II	13	16,766	17,809	1,044	918	2	Glyco_hydro_18
WH10009194	A-II	140	37,55,612	37,56,901	1,290	1,170	2	Glyco_hydro_18
WH10003963	A-IV	44	36,47,885	36,49,057	1,173	1,047	2	Glyco_hydro_18
WH10009310	A-IV	140	40,97,852	40,99,181	1,330	1,080	3	Glyco_hydro_18
WH10000184	A-V	195	7,12,351	7,14,343	1,993	1,137	3	Glyco_hydro_18
WH10004667	A-V	84	16,96,826	16,98,273	1,448	1,263	3	Glyco_hydro_18
WH10004720	A-V	84	19,43,371	19,44,863	1,493	1,440	1	Glyco_hydro_18
WH10008231	A-V	13	53,47,413	53,51,929	4,517	1,365	3	Glyco_hydro_18
WH10008749	A-V	140	22,05,113	22,06,420	1,308	1,191	2	Glyco_hydro_18
WH10006436	B-I	81	27,86,930	27,87,979	1,050	1,050	0	GH18_chitinase_D-like
WH10000865	B-I	394	9,80,828	9,81,829	1,002	1,002	0	GH18_hevamine_XipI_class_III
WH10002591	B-I	19	8,92,234	8,93,241	1,008	954	1	GH18_hevamine_XipI_class_III
WH10003851	B-I	44	27,94,213	27,95,364	1,152	1,095	1	GH18_hevamine_XipI_class_III
WH10003001	B-II	19	33,57,251	33,58,748	1,498	1,173	3	GH18_hevamine_XipI_class_III
WH10004309	B-II	84	6,28,967	6,30,185	1,219	969	4	GH18_hevamine_XipI_class_III
WH10002868	B-V	19	25,31,434	25,32,510	1,077	1,077	0	GH18_CTS3_chitinase
WH10002282	B-V	16	31,24,941	31,25,942	1,002	1,002	0	GH18_CTS3_chitinase
WH10003288	C-I	44	6,07,411	6,11,216	3,806	3,747	1	GH18_chitolectin_chitotriosidase
WH10002109	C-I	16	25,13,862	25,19,348	5,487	4,221	14	GH18_chitolectin_chitotriosidase
WH10009911	C-I	140	60,14,677	60,19,121	4,445	3,528	11	GH18_chitolectin_chitotriosidase
WH10003350	C-I	44	9,30,019	9,32,820	2,802	2,661	2	GH18_zymocin_alpha
WH10005062	C-I	84	30,17,609	30,21,222	3,614	2,565	8	GH18_zymocin_alpha
WH10002350	C-I	16	33,22,645	33,24,428	1,784	1,638	2	GH18_zymocin_alpha
WH10002829	C-I	19	22,66,811	22,68,792	1,982	1,617	6	GH18_zymocin_alpha
WH10005656	C-I	84	50,28,566	50,30,541	1,976	1,596	6	GH18_zymocin_alpha
WH10001817	C-II	16	8,25,397	8,27,224	1,828	1,431	5	GH18_zymocin_alpha
WH10005213	C-II	84	36,11,927	36,13,581	1,655	1,362	5	GH18_zymocin_alpha
WH10001816	C-II	16	8,24,242	8,25,375	1,134	1,134	0	GH18_zymocin_alpha
WH10007469	C-II	13	22,36,163	22,39,948	3,786	3,564	4	GH18_zymocin_alpha
WH10001630	C-II	257	27,17,848	27,22,337	4,490	3,891	8	GH18_zymocin_alpha
WH10001666	C-II	16	2,87,779	2,90,059	2,281	2,040	2	GH18_zymocin_alpha
WH10005780	C-II	81	3,55,979	3,60,265	4,287	3,546	5	GH18_zymocin_alpha
WH10006445	C-II	81	28,86,438	28,90,875	4,438	3,732	5	GH18_zymocin_alpha
WH10010026	C-II	140	66,00,623	66,04,947	4,325	4,113	3	GH18_zymocin_alpha

The gene lengths varied from 1,002 to 5,487 bp, and the coding sequences (CDS) of the genes ranged from 918 to 4,221 bp. The peptide lengths ranged from 305 (WH10006907) to 1406 (WH10002109) amino acids, corresponding to molecular weights (MW) of 33.28 kDa (WH10006907) and 150.78 kDa (WH10002109), respectively. The isoelectric points (*pI*) ranged from 4.21 (WH10001817) to 8.01 (WH10003001) (Table 3).

**TABLE 3:** Protein structure analysis of 41 GH18 genes of highly pathogenic strain Hp10 of *M. perniciosa.*

**Genes**	**Type**	**Length (aa)**	**MW (kDa)**	**PI**	**Instability index**	**Fat index**	**GRAVY**	**Localization predicted**
WH10000025	A-II	342	37.13	6.32	28.45	73.98	–0.193	Extracellular space
WH10000176	A-II	361	39.41	5.72	27.64	75.76	–0.124	Extracellular space
WH10000259	A-II	362	39.44	6.09	29.9	74.75	–0.173	Extracellular space
WH10003018	A-II	370	40.76	5.79	30.43	72.03	–0.272	Organelle membrane
WH10003193	A-II	347	37.88	6.31	27.26	74.03	–0.153	Extracellular space
WH10006636	A-II	362	39.56	5.9	30.79	72.32	–0.184	Extracellular space
WH10006653	A-II	317	35.11	5.11	31.83	66.53	–0.323	Extracellular space
WH10006907	A-II	305	33.28	5.86	31.87	73.31	–0.241	Extracellular space
WH10009194	A-II	389	44.04	5.03	29.75	79.23	–0.299	Extracellular space
WH10003963	A-IV	348	38.41	6.55	37.65	82.39	–0.266	Cytoplasm
WH10009310	A-IV	359	38.83	6.41	38.84	85.65	–0.025	Extracellular space
WH10000184	A-V	378	41.81	4.78	29.66	74.39	–0.396	Cytoplasm
WH10004667	A-V	420	46.15	6.52	28.72	75.57	–0.32	Extracellular space
WH10004720	A-V	479	52.31	5.23	37.22	73.9	–0.365	Extracellular space
WH10008231	A-V	454	50.48	6.27	41.91	69.65	–0.328	Mitochondrion
WH10008749	A-V	396	44.4	4.6	35.81	69.04	–0.564	Cytoplasm
WH10006436	B-I	349	36.8	7.6	25.27	93.15	0.18	Extracellular space
WH10000865	B-I	333	35.32	5.06	31.75	75.38	–0.122	Extracellular space
WH10002591	B-I	317	33.46	6.14	29.96	88.49	–0.077	Extracellular space
WH10003851	B-I	364	39.93	4.89	39.68	65.96	–0.321	Extracellular space
WH10003001	B-II	390	40.89	8.01	39.03	64.64	–0.129	Extracellular space
WH10004309	B-II	322	34.65	6.23	26.6	77.61	–0.025	Extracellular space
WH10002282	B-V	333	37.57	4.96	40.84	84.62	–0.33	Cytoplasm
WH10002868	B-V	358	39.87	6.13	37.26	84.75	–0.156	Extracellular space
WH10003288	C-I	1,248	135.25	5.42	41.39	64.79	–0.411	Mitochondrion
WH10002109	C-I	1,406	150.78	4.86	42.24	71.34	–0.135	Extracellular space
WH10009911	C-I	1,175	130.16	6.76	32.1	81.5	–0.216	Extracellular space
WH10003350	C-I	886	100.33	6.19	45.42	66.93	–0.519	Cytoplasm
WH10005062	C-I	854	96.48	6.68	33.94	63.1	–0.564	Cytoplasm
WH10002350	C-I	545	58.57	4.92	36.2	74.84	–0.045	Plasma membrane
WH10002829	C-I	538	59.3	5.42	36.78	69.52	–0.28	Endomembrane system
WH10005656	C-I	531	58.47	5.68	34.73	64.75	–0.321	Plasma membrane
WH10001817	C-II	476	51.16	4.21	42.1	69.03	–0.199	Extracellular space
WH10005213	C-II	453	50.71	4.66	41.42	70.02	–0.518	Cytoplasm
WH10007469	C-II	1,187	124.89	5.99	48.14	73.84	–0.003	Extracellular space
WH10001630	C-II	1,296	138.06	6.11	34.28	75.69	–0.089	Extracellular space
WH10001666	C-II	679	73.67	6.7	38.77	74.93	–0.225	Extracellular space
WH10001816	C-II	377	39.96	4.44	36.06	72.52	–0.158	Extracellular space
WH10005780	C-II	1,181	128.06	5	31.36	73.29	–0.32	Extracellular space
WH10006445	C-II	1,243	135.6	5.7	33.38	76.28	–0.288	Plasma membrane
WH10010026	C-II	1,370	149.77	6.07	32.21	72.07	–0.325	Extracellular space

The grand average of hydrophobicity (GRAVY), the fat index, and the instability index of the GH18 genes ranged from −0.564 (10008749) to 0.18 (WH10006436), 63.1 (WH10005062) to 93.15 (WH10006436), and 25.27 (WH10006436) to 48.14 (WH10007469), respectively. The amino acid composition and content analysis showed 13 different kinds of amino acids, and glycine and alanine were the predominant residues. The proteins had variable number disulfide bonds and protein-protein binding regions ranging from 1 to 50 and 5 to 37, respectively. All the genes were predicted to have α-helix (6.72–34.12%), β-fold (8.39–25.68%), and random coil (29.84–71.88%) structures. Most of the GH18 proteins (27, 65.85%) were predicted to be located in the extracellular space (matrix), but some proteins were located in the cytoplasm, plasma membrane, mitochondrion, endomembrane system, and organelle membranes. The amino acid peptide length, molecular weight, isoelectric point, instability index, fat index, GRAVY, and putative *in silico* subcellular localization predictions of GH18 identified in *M*. *perniciosa* Hp10 are listed in Table 3.

### Phylogenetic Analysis of GH18 Family Proteins

**FIGURE 2:**
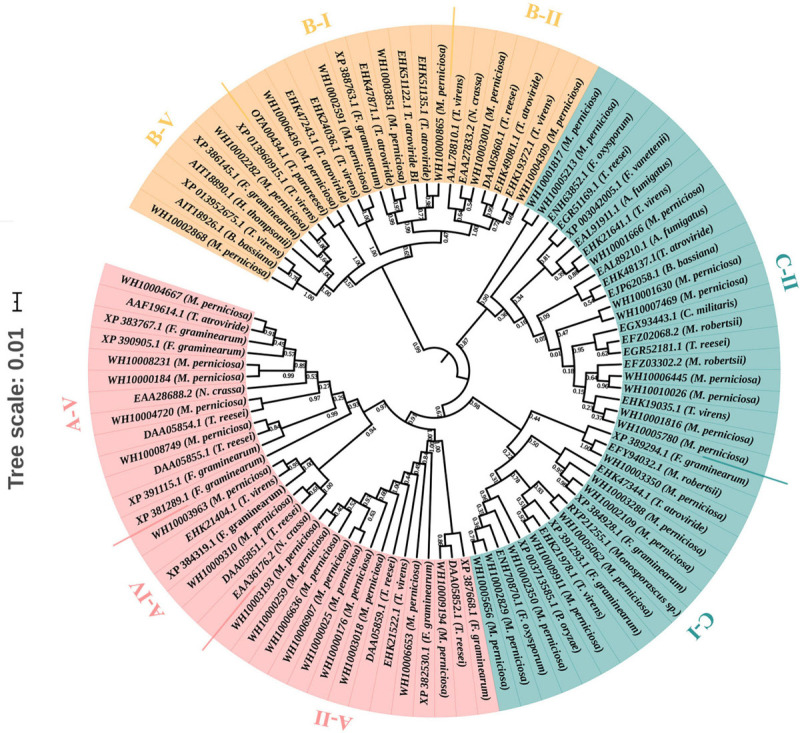
Phylogenetic tree of GH18 genes. Different colors indicate different groups. Red color represents group A. It contains A-II, A-IV, and A-V subgroups. Orange color represents group B. It contains B-I, B-II, and B-V subgroups. Green color r represents group C, and it contains C-I and C-II subgroups.

To predict the functions and better understand the evolutionary relationships of GH18 chitinase proteins among different species, a phylogenetic tree was constructed using the 41 GH18 chitinase protein sequences of *M*. *perniciosa* Hp10 and 56 GH18 protein sequences of 15 other fungal species. The phylogenetic tree clustered the 41 GH18 chitinase protein sequences of *M*. *perniciosa* Hp10 into three major groups (A, B, and C) and eight different subgroups (A-II, A-IV, A-V; B-I, B-II, and B-V; C-I and C-II) according to sequence similarity of their GH18 catalytic domains (Figure 2). This result was consistent with that using the conserved domains.

### Gene Structure, Conserved Motif Analyses, and Chromosomal Location

The exon-intron structure was analyzed to provide further insight into the evolution of the GH18 genes. The numbers of exons/introns in the GH18 gene family ranged from 1 to 15 and 0 to 14, respectively. Although GH18 genes with high similarity were clustered in the same group, the numbers, distribution, and locations of the exons/introns were different (Figure 1B). Group C contained the highest numbers of exons/introns (1–15 exons and 0–14 introns), and Group B had the lowest numbers of exon and intron (1–5 exons and 0–4 introns). Fifteen distinct motifs with sizes ranging from 15 to 50 amino acids were identified among the 41 GH18 proteins (Figure 1C). From the Pfam analysis, 11 out of the 15 proteins encoded functional domains. Except for motif 9, which encoded chitin recognition protein, the rest of the motifs (1–5, 7–8, 12, 14, and 15) with functional domains encoded glycosyl hydrolases family 18. Motif 1, followed by motifs 4, 12, and 2, were widely distributed in all of the GH18 genes (31, 30, 30, and 29 genes, respectively). Also, most GH18 proteins in the same group or subgroup shared similar motif types and distributions. For example, except for WH10002282, all the members of groups B, D, and E contained two motifs (motifs 2 and 10).

**FIGURE 3:**
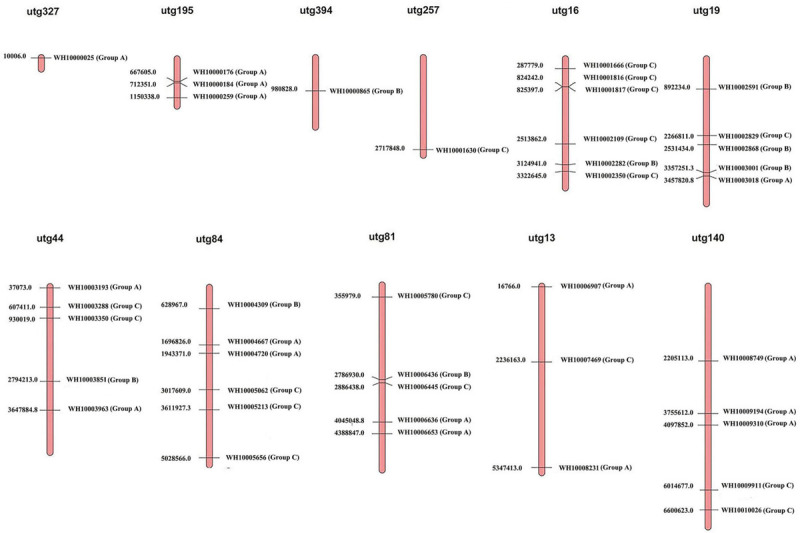
Distribution of 41 GH18 genes on *M. perniciosa* Hp10 utgs.

The 41 GH18 genes were distributed across 11 scaffolds out of the 23 scaffolds of the *M*. *perniciosa* Hp10 genome (Figure 3). Scaffolds utg16, utg81, and utg84 contained the highest number (6) of GH18 genes each (accounting for 44% of genes mapped), while the lowest number (1) of GH18 genes was found on scaffolds utg327, utg394, and utg257. In addition, utg16 and utg81 each had four and three GH18 genes with chitin-binding domain LysM or chtbd1. Scaffolds (utg19 and utg140), (utg44), and (utg13 and utg195) contained five, four, and three GH18 genes, respectively.

### Identification of Orthologs and Gene Duplication

A total of 35 orthologous clusters were identified among the 16 fungal species. All 41 genes of *M*. *perniciosa* Hp10 formed orthologous groups with 13 fungal species (Supplementary S1). In total, 180 gene duplication events were identified within the species tree. Twelve gene duplication events were identified in *M*. *perniciosa* Hp10, and these were located on terminal branches of the species tree. Eight genes (WH10000176 and WH10000184, WH10001816 and WH10001817, WH10003001 and WH10002868, and WH10006436 and WH10006445) appeared as tandem repeats on utg195, utg16, utg19, and utg18, but there were not tandem array genes. The estimated Ka/Ks ratios of the genes in the twelve duplication events varied between 0.3842 and 3.5969. The estimated Ka/Ks ratios of four gene duplication events were above 1 (Ka/Ks > 1), whereas the other eight gene duplication events were below 1 (Ka/Ks < 1) (Supplementary S2).

### Expression Profile of *M. perniciosa* Hp10 During Infection of *A*. *bisporus*

**FIGURE 4:**
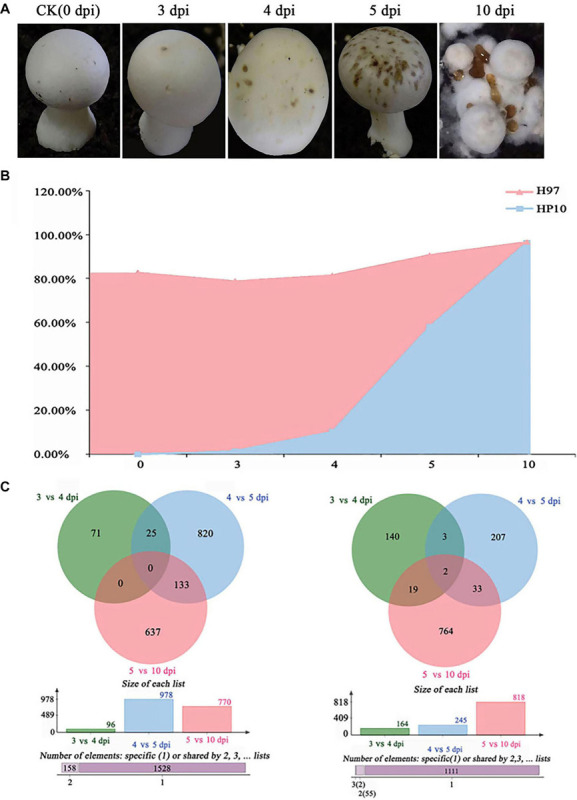
Transcriptome analysis of different stages of *M. perniciosa-*infected *A. bisporus.* **(A)** Cap symptoms in different infection periods (0, 3, 4, 5, and 10 dpi). **(B)** The clean reads were mapped to both the pathogen Hp10 and the reported host *A. bisporus* H97 genomes. **(C)** Differential expression of genes in different infection stages; the former one represents upregulation and the latter one represents downregulation.

To investigate the gene expression of *M*. *perniciosa* Hp10, we performed RNA-seq profiling of *M*. *perniciosa* Hp10 at 0, 3, 4, 5, and 10 days post-inoculation (dpi) during the infection of *A*. *bisporus* (Figure 4A). In total, 78.64 Gb of high-quality clean reads was generated after raw data filtering and trimming, with a Q20 quality score ≥97%. The transcriptome sequencing depths were identified as being close to saturation. The clean reads were mapped to both the pathogen *M*. *perniciosa* Hp10 and the host *A. bisporus* H97 genomes (Figure 4B). The results revealed that the proportions of total clean reads mapped to the pathogen *M*. *perniciosa* Hp10 reference genome were 0.03, 1.45, 10.15, 57.92, and 96.22% at 0, 3, 4, 5, and 10 dpi, respectively. The proportions increased with the post-infection times, reflecting the increase in production of the pathogenic fungal biomass. For the mapping to the host *A. bisporus* H97 reference genome, the proportions were 82.48, 77.19, 71.18, 32.63, and 0.27% at 0, 3, 4, 5, and 10 dpi, respectively, indicating a decrease with the post-infection times.

Therefore, we selected four infection stages (3, 4, 5, and 10) to map to the pathogen *M*. *perniciosa* Hp10 genome to perform further identification of the pathogenicity-related genes. The normalized differentially expressed genes (DEGs) were calculated by comparisons between different time points using the log2 fold change (| log2FC [fold change] | > 1, *p* < 0.005).

Overall, 8,425 (78.68%) genes were expressed in all samples during the infection process. Among these, 5441 DEGs were annotated using the GO, KEGG, and PFAM databases (Supplementary S3). There were 3,071 (30.47%) genes that exhibited significant differential expression. Many of the significant DEGs encoded known proteins (99%). In total, 1,844 (17.22%) genes were upregulated and 1,227 (11.46%) genes were downregulated (Figure 4C) relative to the comparisons at different time points (3 vs. 4 dpi; 4 vs. 5 dpi; and 5 vs. 10 dpi) in *M*. *perniciosa* Hp10.

The results showed that 96 genes were upregulated and 164 genes were downregulated at 3 vs. 4 dpi, while at 4 vs. 5 dpi and 5 vs. 10 dpi, 978 and 770 genes were upregulated and 245 and 818 genes were downregulated, respectively. The fold change (log2 ratio) of the gene expression ranged from -5.26 to 4.17, -7.55 to 10.1, and -11.39 to 15.01 for 3 vs. 4 dpi, 4 vs. dpi, and 5 vs. 10 dpi, respectively. We identified 25 upregulated DEGs shared between 3 vs. 4 dpi and 4 vs. 5 dpi, and 133 upregulated DEGs were shared between 4 vs. 5 dpi and 5 vs. 10 dpi. We also identified 71, 820, and 637 upregulated DEGs unique to 3 vs. 4 dpi, 4 vs. 5 dpi, and 5 vs. 10 dpi, respectively.

The significantly upregulated and downregulated genes were functionally annotated using the GO, KEGG, and PFAM databases. The GO enrichment categories were classified to biological process (50.85%, 1,585 genes), molecular function (32.63%, 1,017 genes), and cellular component ontology (16.52%, 515 genes). The most enriched upregulated genes for 3 vs. 4 dpi were annotated to transmembrane transport (BP), an integral component of membrane (CC), and phosphatidylserine decarboxylase activity. For 4 vs. 5 dpi and 5 vs. 10 dpi, the most enriched upregulated genes were annotated to oxidation-reduction process, an integral component of membrane, oxidoreductase activity, translation regulation fungal-type membrane, and heme-binding. None of the 3 vs. 4 dpi downregulated genes were enriched. Mycoparasitism-related genes, including transporters (major facilitator superfamily (MFS), ATP-binding cassette (ABC), sugar and phosphate transporter), peptidases, carbohydrate-active enzymes, cytochrome P450 monooxygenases (CYP), polyketide synthases (PKS), WD40 proteins, and transcription factors (CCHC zinc finger, fungal-specific transcription factor domain, and Zn (2) Cys (6) transcription factors), were highly upregulated at all of the time points. Through the statistical analysis, we found that the functions of the most significant DEGs were related to transporters, transporter regulators, pathogenicity, peptidase, stress, secondary metabolites, and mating (Table 4).

**TABLE 4:** The most significant DEGs in different infection stages.

**Infection stage**	**Protein ID**	**Putative function**	***P*-value**	***Q-*value**	**Expression patterns**
3 vs. 4 dpi	WH10001910	Sugar (and other) transporter	4.77E-10	8.29E-07	Upregulated
4 vs. 5 dpi	WH10006740	AMP-binding enzyme	3.62E-13	4.12E-12	Upregulated
4 vs. 5 dpi	WH10005671	Ankyrin repeats (3 copies)	1.83E-10	1.7E-09	Upregulated
4 vs. 5 dpi	WH10000037	ANTH domain	6.4E-16	8.76E-15	Upregulated
4 vs. 5 dpi	WH10009928	Carbamoyl-phosphate synthase L chain, N-terminal domain	2.47E-44	1.01E-42	Upregulated
4 vs. 5 dpi	WH10000866	Carbohydrate-binding family 9	3.7E-61	2.94E-59	Upregulated
4 vs. 5 dpi	WH10000743	CFEM domain	5.68E-38	1.89E-36	Upregulated
4 vs. 5 dpi	WH10009972	Cytochrome P450	1.09E-34	3.29E-33	Upregulated
4 vs. 5 dpi	WH10000403	Fungal specific transcription factor domain	0.0000817	0.0003945	Upregulated
4 vs. 5 dpi	WH10000342	Glycosyl hydrolase family 92	3.38E-25	7.36E-24	Upregulated
4 vs. 5 dpi	WH10002227	Heterokaryon incompatibility protein (HET)	0.016321942	0.0474687	Upregulated
4 vs. 5 dpi	WH10009927	LamB	1.18E-25	2.61E-24	Upregulated
4 vs. 5 dpi	WH10006445	LysM domain	3.55E-40	1.24E-38	Upregulated
4 vs. 5 dpi	WH10001714	Major Facilitator Superfamily	0.004681443	0.015798	Upregulated
4 vs. 5 dpi	WH10002283	Mechanosensitive ion channel	1.33E-12	1.45E-11	Upregulated
4 vs. 5 dpi	WH10000836	N-terminal domain of NWD NACHT-NTPase	0.00000985	0.0000546	Upregulated
4 vs. 5 dpi	WH10006568	Peptidase inhibitor I9	1.75E-171	8.07E-169	Upregulated
4 vs. 5 dpi	WH10005697	Phosphate transporter family	5.93E-51	3.23E-49	Upregulated
4 vs. 5 dpi	WH10008274	short chain dehydrogenase	3.56E-50	1.88E-48	Upregulated
4 vs. 5 dpi	WH10010023	WSC domain	0.000694871	0.0028338	Upregulated
4 vs. 5 dpi	WH10004197	Zinc-binding dehydrogenase	2.24E-48	1.09E-46	Upregulated
5 vs. 10 dpi	WH10008279	Acetyltransferase (GNAT) domain	5.46E-26	1.73E-24	Downregulated
5 vs. 10 dpi	WH10008187	ANTH domain	1.03E-51	1.14E-49	Upregulated
5 vs. 10 dpi	WH10003199	ATPase family associated with various cellular activities (AAA)	1.09E-18	2.18E-17	Downregulated
5 vs. 10 dpi	WH10003223	Beta-ketoacyl synthase, N-terminal domain	0.000168808	0.0006965	Downregulated
5 vs. 10 dpi	WH10008230	Cyclin, N-terminal domain	5.85E-45	4.86E-43	Downregulated
5 vs. 10 dpi	WH10003021	Cytochrome P450	3.25E-104	1.45E-101	Downregulated
5 vs. 10 dpi	WH10005692	FAD binding domain	1.02E-85	2.87E-83	Downregulated
5 vs. 10 dpi	WH10000259	Glycosyl hydrolases family 18	1.44E-30	6.41E-29	Downregulated
5 vs. 10 dpi	WH10000139	Indoleamine 2,3-dioxygenase	5.95E-19	1.22E-17	Downregulated
5 vs. 10 dpi	WH10006445	LysM domain	4.38E-65	6.93E-63	Downregulated
5 vs. 10 dpi	WH10003020	Major facilitator superfamily	8.58E-66	1.38E-63	Downregulated
5 vs. 10 dpi	WH10002881	Meiotically up-regulated gene family	2.42E-18	4.71E-17	Downregulated
5 vs. 10 dpi	WH10002918	Acyl transferase domain	4.23E-35	2.36E-33	Upregulated
5 vs. 10 dpi	WH10001707	Chitin synthase	1.21E-184	1.18E-180	Upregulated
5 vs. 10 dpi	WH10002685	Cytochrome P450	1.39E-31	6.57E-30	Upregulated
5 vs. 10 dpi	WH10003006	Matrixin	0.01505043	0.038037	Upregulated
5 vs. 10 dpi	WH10002449	GppA phosphatase family	0.001327168	0.0044838	Upregulated
5 vs. 10 dpi	WH10009603	Ring finger domain	3.97E-33	2.04E-31	Upregulated

### Expression Pattern of GH18 Genes During *M. perniciosa* Hp10 Infection of *A. bisporus*

**FIGURE 5:**
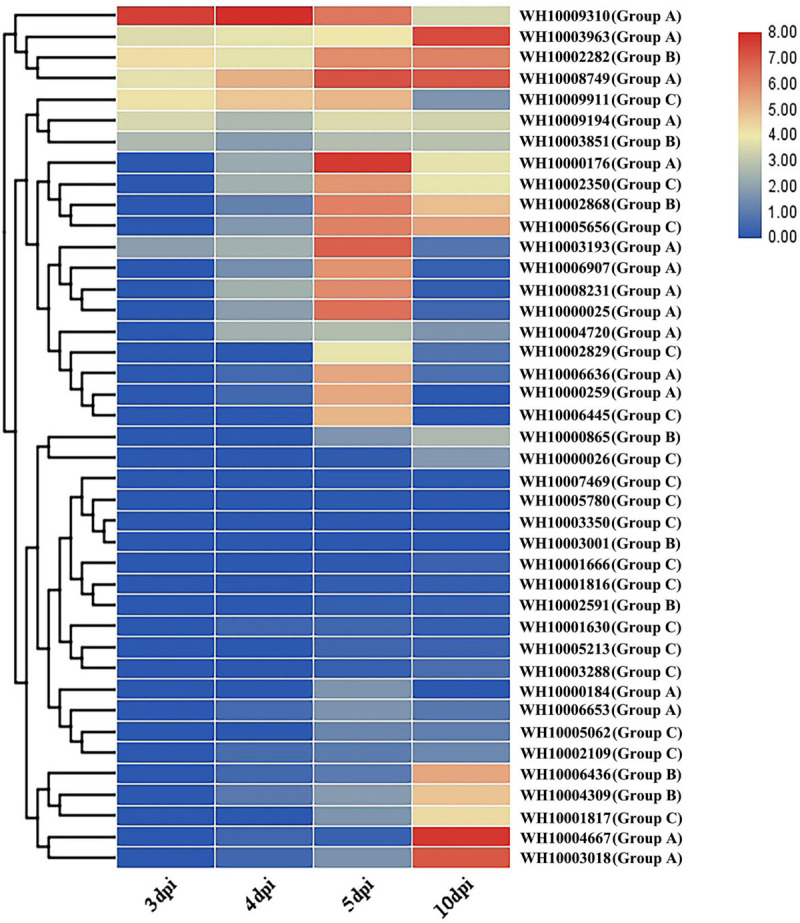
Gene expression profile of the chitinases GH18 gene family of *M. perniciosa* Hp10.

To understand the possible roles of *M*. *perniciosa* GH18 genes in *A*. *bisporus*, we characterized the 41 GH18 genes expressed during the disease infection period. The results showed that 23 genes out of the 41 GH18 genes were differentially expressed during the disease infection stage (Figure 5). The results showed no differential expression of GH18 genes at 3 vs. 4 dpi, while 15 GH18 genes were differentially expressed at 4 vs. 5 dpi, and only one gene (WH10009310) was significantly downregulated. The other 14 genes were significantly upregulated, including eight genes in group A (WH10000025, WH10000176, WH10000259, WH10003193, WH10006636, WH10006907, WH10008231, and WH10008749), two genes in group B (WH10002282 and WH10002868), and four genes in group C (WH10002350, WH10002829, WH10005656, and WH10006445). At 5 vs 10 dpi, 13 genes were downregulated, including nine genes in type A (10000025, WH10000176, WH10000184, WH10000259, WH10003193, WH10006636, WH10006907, WH10008231, and WH10009310) and four genes in group C (WH10002350, WH10002829, WH10006445, and WH10009911); six genes had upregulated expression levels, including three genes in type A (WH10003018, WH10003963, and WH10004667), two genes in group B (WH10004309 and WH10006436), and one gene in group C (WH10001817).

### Gene Cloning and Analysis of GH18 Genes of *M. perniciosa* Hp10

**FIGURE 6:**
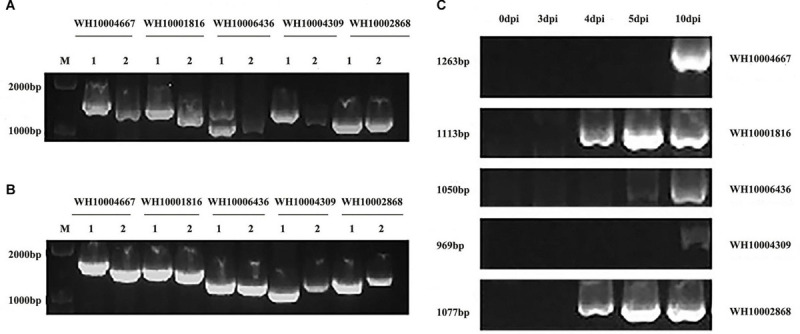
Electrophoretogram of gene cloning and RT-PCR verification of five GH18 genes. **(A)** DNA and cDNA of the five GH18 genes. **(B)** Identification of recombinant cloning vectors of five GH18 genes. **(C)** Expression of five GH18 genes in different infection periods. (1) DNA. (2) cDNA.

Five representative GH18 genes from groups A, B, and C (WH10004667, WH10004309, WH10002868, WH10006436, and WH10001816) (Figure 6) were cloned and sequenced to verify the accuracy of the genome and transcriptome analyses. The results showed that each of the target gene lengths of DNA and cDNA sequences was 1,000–1,500 bp and 1,000–1,300 bp, respectively. The genes showed 100% similarity to the sequences used for the *in silico* analysis. In addition, the CDS length (969–1,263 bp), amino acids (322–420), and introns (0–4) of conserved domains indicated that these were GH18 genes. From the RT-PCR results, we found that these five genes were involved in the infection process, and some genes were differentially expressed; likely WH10004667, WH10004309, and WH10006436 were upregulated at 5 vs. 10 dpi, while WH10002868 was upregulated at 4 vs. 5 dpi, consistent with the transcriptome analysis.

## Discussion

Despite the economic importance of wet bubble disease of *A*. *bisporus*, the details of the genes expressed by *M*. *perniciosa* during infection of its host remain largely unknown. In our previous work, we sequenced the genome of *M*. *perniciosa* and identified 41 GH18 chitinase genes and putative genes related to pathogenicity (Li et al., 2019). The GH18 chitinases are diverse multigene families in a wide range of organisms and are known to play essential roles in biological processes like growth, nutrient acquisition, interspecific interactions, pathogenesis, and defense (Gruber and Seidl-Seiboth, 2012; Nagpure et al., 2013).

In this study, we performed deep genome mining and characterized a total of 41 GH18 genes in *M*. *perniciosa* Hp10. Furthermore, we analyzed the transcriptome profile of *M*. *perniciosa* Hp10 and the expression patterns of its GH18 genes during infection of *A*. *bisporus*. The 41 GH18 proteins’ physicochemical characteristics were similar to those of other filamentous fungi, and the genes had detectable transcripts, thereby validating their functionality. However, the number of GH18 genes in *M*. *perniciosa* Hp10 was higher than those in *F. graminearum*, *M. oryzae*, *N. crassa*, *R. solani*, and *T. virens* (Xue et al., 2012; Zhang et al., 2014; Zapparata et al., 2017), which generally range from 10 to 30 genes. The high number of GH18 genes in *M*. *perniciosa* Hp10 is characteristic of mycoparasitic fungi (*T. atroviride* and *T. virens*), as it may require several chitinase isozymes acting in synergy to enable degradation of the host chitin cell wall (Lichius et al., 2014).

The *pI* analysis revealed that most of the GH18 chitinases are acidic enzymes, except two genes that encode alkaline chitinases (Table 3), consistent with the results of previous studies (Seidl et al., 2005). The different subcellular localization patterns of the GH18 proteins suggest that they might be differentially regulated and may have distinct roles in *M*. *perniciosa* Hp10. The GH18 proteins secreted into the extracellular space might function as virulence factors and in cell wall remodeling (Zamith-Miranda et al., 2018; Vincent et al., 2019). The finding also suggests that the proteins could be employed as drug targets to control *M*. *perniciosa* (Blum et al., 2009). The subcellular localization of proteins is invaluable for understanding their functions and interactions with other proteins (Peng and Gao, 2014).

Phylogenetic analysis revealed that the 41 *M*. *perniciosa* Hp10 GH18 genes were clustered into eight subgroups among three groups (A, B, and C), a result that is consistent with the previous classification of GH18 gene families in related genera (Seidl et al., 2005; Karlsson and Stenlid, 2008). The numbers of genes in groups A, B, and C were higher than those reported for *Trichoderma* species (Kubicek et al., 2011). In addition, *M*. *perniciosa* Hp10 GH18 genes in the same group and subgroup had a similar conserved domain and motif distributions to closely related members in the phylogenetic tree, revealing the functional similarity among the same subgroup proteins. Gene structure analysis of the clustered groups showed variation in the intron and exon numbers and lengths, but all had conserved motifs. In general, genes in group C had more introns than those in group A and B. A small number of introns in a gene is the result of genetic evolution and can help the gene be rapidly regulated during a stress response, and the introns also serve as a source of sequence variation (Jacob and Smith, 2017; Naro and Sette, 2017). At the same time, intron retention may also increase the complexity of this protein family (Zhang et al., 2004). Orthology analysis revealed that all the *M*. *perniciosa* Hp10 GH18 genes were homologous to the GH18 genes of the 13 species used in the phylogenetic tree. This suggests that *M*. *perniciosa* Hp10 GH18 genes and those of the other 13 species evolved from a common ancestor.

*M*. *perniciosa* Hp10 Group A (and subgroups) GH18 genes displayed extensive homology to *T. virens* endo- and exo-chitinases, and they play a role in self- and non-self-cell wall degradation (Gruber and Seidl-Seiboth, 2012) and mycoparasitism (Tzelepis et al., 2015). The group B chitinase were homologous to subgroup B endochitinases and subgroup B-V [endo-beta-N-Acetylglucosaminidase (ENGase)] of *Trichoderma* sp. (Seidl et al., 2005; Tzelepis et al., 2014) and *B. bassiana*, *F. graminearum*, *H. thompsonii*, and *T*. *virens* (Lichius et al., 2014). *M*. *perniciosa* Hp10 group B GH18 proteins are involved in cell wall synthesis and remodeling (Tzelepis et al., 2015), and those with CBM increase the hydrolysis of insoluble substrates (Limón et al., 2001). All the group C GH18 proteins were similar to *T*. *virens* group C proteins. Each has a structure composed of a class 1 chitin-binding domain (CBM18) (Wright et al., 1991), including cysteine linked by eight disulfide bonds (Viterbo et al., 2001), and accompanied by one or two LysM domains. Group C displayed similarity to yeast killer toxin and may be involved in a killer-toxin-like mechanism of permeabilizing antagonist cell walls in fungal–fungal interactions (Gruber et al., 2011a,b; Tzelepis et al., 2012; Tzelepis and Karlsson, 2019). The *M*. *perniciosa* Hp10 group C GH18 genes are speculated to be involved in nutrition, hyphal growth and development, fungal-fungal interactions, and virulence to host fungi (Dana et al., 2001; Baratto et al., 2006; Kubicek et al., 2011; Seidl-Seiboth et al., 2013).

The GH18 genes were unevenly distributed on different contigs, and two gene pairs showed tandem repeats on four different contigs. Further analysis revealed the duplication of *M*. *perniciosa* Hp10 GH18 genes. We found that the majority of the GH18 genes of 13 species were arranged tandemly. This suggests that tandem duplication has been a major process in the evolution of the GH18 gene family through unequal crossover events (Gruber et al., 2011a). The duplication of genes allows the accumulation of mutations (on independent replication of a single sequence), leading to an increase of divergence and subsequent expansion of the gene family (Bowers et al., 2003; Gu et al., 2003, 2005). The Ka/Ks ratios revealed that *M*. *perniciosa* GH18 genes had undergone the process of both positive selection (Ka/Ks > 1) and purifying selection (Ka/Ks < 1). GH18 genes in *Trichoderma* species have been reported to evolve under positive, neutral, and purifying selections (Ihrmark et al., 2010). The positive selection might change the protein structure and increase the functional divergence of the enzyme, whereas the purifying selection suggests the GH18 proteins preserve the ancestral function of duplicated genes (Ihrmark et al., 2010; Kubicek et al., 2011).

The transcriptome analysis of different stages of *M. perniciosa-*infected *A. bisporus* revealed that a tiny percentage of clean reads was mapped to the *M. perniciosa* genome at 1–3 dpi (less than 2%), indicating the low levels of biomass during this period of *A. bisporus* basidiome colonization (Suberkropp, 2001; Rudd et al., 2015). From the onset of symptoms 4–10 dpi, there was an increased proportion of reads mapped to the pathogen genome (i.e., from 10.12 to 96.22%), which could be attributed to the growth of the fungus completely colonizing the host and the substantial suppression or near destruction of the host RNA by the pathogen (Lai et al., 2014; Rudd et al., 2015). There were some highly upregulated DEGs (96) during 3 vs. 4 dpi; at this stage, the conidia had already attached to the host, and the rapid growth of mycelia was underway. The pathogen uses minimal amounts of host-derived nutrients for the initial phase of colonization. Therefore, there is a need for the rapid growth of the intracellular membrane and transport of components in order for the pathogen to acquire nutrients from the host environment (Xie et al., 2014). At 4 vs. 5 dpi, *M*. *perniciosa* Hp10 (Zhang et al., 2017b) overcomes the host defense system and utilizes the host as a source of nutrients, similar to the nutrition style of *Hirsutella minnesotensis* and *Trichoderma* species (Lai et al., 2014; Xie et al., 2014).

*M*. *perniciosa* Hp10 overcompensates for the host defense by producing more peptidases (such as subtilase, metallopeptidase, lipases, and peptidase family M28), and more glycosyl hydrolases, especially GH 18 and LysM domain and other chitin or carbohydrate-modifying or shielding proteins, such as CFEM (Common in several Fungal Extracellular Membrane proteins) and the WSC (Wall Stress-responsive Component) domain, which protects the pathogen, as well as genes for adaptation to environmental stress (Hsp70 protein) (Kubicek and Druzhinina, 2013; Karlsson et al., 2017). The cerato-platanin and FAD-binding proteins of *M*. *perniciosa* Hp10 may be associated with hyphal growth and mycoparasitism, as observed in *T*. *harzianum* (Gomes et al., 2015; Karlsson et al., 2017). The upregulation of the velvet factor mediates the synthesis of secondary metabolites and initiates sexual reproduction and the production of spores and conidia by regulation of meiotically upregulated genes (Lind et al., 2016).

At 10 dpi, many candidate-effector proteins, protein kinases, secondary metabolites, toxins, sexual reproduction genes [HMG (high mobility group) box and Pheromone A receptor], CAZymes, and transcription factors [CHY zinc finger, Zn (2) Cys (6) and bZIP] (Morán-Diez et al., 2015; Karlsson et al., 2017; Druzhinina et al., 2018) were highly expressed. We suggest these DEGs are associated with the pathogen reproduction and proliferation in the host after the defeat of the host tissues. The high upregulation of sexual reproduction genes is necessary for the pathogen to produce a higher number of conidia in order to persist in compost and for dispersal to continue throughout the infection cycle during subsequent flushes of *A*. *bisporus*.

Gene expression analysis showed that 23 of the 41 chitinase GH18 genes of *M. perniciosa* Hp10 were differentially expressed at different infection stages; 20 genes were significantly upregulated and 16 genes were significantly downregulated during the infection process, indicating that chitinase GH18 genes play different roles at different infection stages. The expression of chitinase genes was significantly different at different infection stages, and the number of chitinase genes with differential expression was less at 3 vs. 4 dpi, but 14 genes were significantly upregulated at 4 vs. 5 dpi, indicating that these 14 genes play important roles in the process of *M. perniciosa* infecting *A. bisporus*. Six genes were significantly upregulated at 5 vs. 10 dpi, indicating that these genes are closely related to the pathogen’s growth and development and its mass reproduction in the host. The proportions and types of chitinase genes significantly upregulated in different infection or development stages were different, indicating that the chitinase GH18 gene family has undergone functional differentiation during evolution. The genes belonging to different types may have different functions.

In order to verify the accuracy of genome sequencing and to further analyze the function of the chitinase gene family of *M. perniciosa*, five typical genes were cloned in this study. It was found that all five GH18 genes had α helix structures, β folding structures, and irregular curling structures. The proportion of irregular curling to amino acids of each gene was the largest, while the proportion of α helix and β folding structure to amino acids of each gene was relatively small. In addition, the lengths of these five GH18 genes are all within 1,500 bp, but 322–420 amino acids can be encoded. It is speculated that different RNA splicing methods participate in the expression of chitinase genes, and more chitinase or other proteins can be expressed in the different infection processes and in different host types, allowing the pathogen to expand its host range (Li et al., 2019).

## Conclusion

In summary, a total of 41 GH18 genes were identified in *M*. *perniciosa* Hp 10, with variation in gene structure, protein length, and physicochemical properties. A total of 12 gene duplication events were observed in the GH18 genes and were under positive gene selection. The transcriptome analysis during the infection of *M*. *perniciosa* Hp10 infection of *A*. *bisporus* revealed that the expression of diverse genes, including those coding for CAZyme, proteases, peptidases, effectors, P450, secondary metabolites, and transcription factors, was involved in pathogenicity and the interaction of the pathogen with its fungal host. The expression patterns of 23 GH18 genes differentially expressed at different infection stages were analyzed, and RT-PCR indicated that the GH18 gene family plays an important role in the infection process. The genes identified by the transcriptome analysis will be valuable targets for functional characterization of the potential pathogenicity factors underlying the molecular mechanism of *M*. *perniciosa*, causing wet bubble disease of *A*. *bisporus*. Understanding the strategies employed by *M*. *perniciosa* to infect *A*. *bisporus* and the host’s response to the pathogen can serve as the basis for developing efficient disease-management strategies to mitigate wet bubble disease.

## Data Availability Statement

The required data type (RNA-seq data) in our manuscript has been submitted to the NCBI SRA database (https://www.ncbi.nlm.nih.gov/sra/?term = SRP190007).

## Author Contributions

DL, SX, and YL: conceptualization and funding acquisition. YY and FLS: writing original draft and resources. ZL, YF, XY, KDH, and SX: writing review and editing. YY, YF, and DL: formal analysis. FLS, YF, XY, and SX: software. YY, FLS, ZL, YF, XY, KDH, and DL: methodology. All authors contributed to the article and approved the submitted version.

## Conflict of Interest

The authors declare that the research was conducted in the absence of any commercial or financial relationships that could be construed as a potential conflict of interest.

## Footnotes

1 https://www.uniprot.org/uniprot/

2 https://www.ebi.ac.uk/interpro/

3 http://hmmer.janelia.org/

4 http://smart.emblheidelberg.del

5 https://www.ncbi.nlm.nih.gov/Structure/cdd/wrpsb.cgi/

6 https://web.expasy.org/protparam/

7 http://busca.biocomp.unibo.it/

8 http://gsds.cbi.pku.cn/index-php

9 http://meme-suite.org/tools/meme

10 https://www.predictprotein.org/

11 https://www.swissmodel.expasy.org/

12 http://www.nipgr.res.in/ngsqctoolkit.html

13 http://ccb.jhu.edu/software/tophat/index.shtml

14 http://www.kegg.jp/kegg/kegg1.html

## Abbreviations

dpi: day(s) post-inoculation
GH: glycosyl hydrolase
GRAVY: grand average of hydropathicity
MW: molecular weight
NJ: neighbor-joining
PI: isoelectric point
RT-PCR: reverse-transcription polymerase chain reaction
WBD: wet bubble disease.
