# Supplementary material for: Genome-Wide Identification and Analysis of Chitinase GH18 Gene Family in Mycogone perniciosa
Source: Front Microbiol. 2021 Jan 11;11:596719. doi: 10.3389/fmicb.2020.596719 (PMC7829358; doi:10.3389/fmicb.2020.596719)
Supplement: Supplementary file 1 [file Table_1.docx]

Table 1 Oligonucleotide primers for gene cloning

| **Primer** | **Sequence(5'to3')** | Number(bp) |
| --- | --- | --- |
| LD01F | ATGCTCGGTTTTCTCACCAAGT | 22 |
| LD01R | TTAGTTCAGACCGTTCTTGATGTT | 24 |
| LD02F | ATGCGTTCCTCAATGCTC | 18 |
| LD02R | TCACGACAGCGATTCAAC | 18 |
| LD03F | ATGACACGTCTTCTCGAAG | 19 |
| LD03R | TCAGAGCCCGAGCCGC | 16 |
| LD04F | ATGGTTCGCTCTTTGGCTTCT | 21 |
| LD04R | TTAGTTGAGATAGCCGACA | 19 |
| LD05F | ATGAAGTCCCTGTTCCTAT | 19 |
| LD05R | CTATGCATTCACCATTGCC | 19 |
