# Supplementary material for: Genome-Wide Identification and Analysis of Chitinase GH18 Gene Family in Mycogone perniciosa
Source: Front Microbiol. 2021 Jan 11;11:596719. doi: 10.3389/fmicb.2020.596719 (PMC7829358; doi:10.3389/fmicb.2020.596719)
Supplement: Supplementary file 2 [file Table_2.docx]

**Table 2** Gene sequence analysis of 41 GH18 genes of *M. perniciosa* Hp10

| **Genes** | **Group** | **Location** | | | **Gene（bp）** | **CDS (bp)** | **Introns number** | **Conserved domains** |
| --- | --- | --- | --- | --- | --- | --- | --- | --- |
|  |  | **Utg** | **Start** | **End** |  |  |  |  |
| WH10000025 | A-II | 327 | 10006 | 11300 | 1295 | 1029 | 4 | Glyco_hydro_18 |
| WH10000176 | A-II | 195 | 667605 | 668886 | 1282 | 1086 | 3 | Glyco_hydro_18 |
| WH10000259 | A-II | 195 | 1150338 | 1151632 | 1295 | 1089 | 3 | Glyco_hydro_18 |
| WH10003018 | A-II | 19 | 3457821 | 3459136 | 1316 | 1113 | 3 | Glyco_hydro_18 |
| WH10003193 | A-II | 44 | 37073 | 38367 | 1295 | 1044 | 4 | Glyco_hydro_18 |
| WH10006636 | A-II | 81 | 4045049 | 4046343 | 1295 | 1089 | 3 | Glyco_hydro_18 |
| WH10006653 | A-II | 81 | 4388847 | 4390221 | 1375 | 954 | 5 | Glyco_hydro_18 |
| WH10006907 | A-II | 13 | 16766 | 17809 | 1044 | 918 | 2 | Glyco_hydro_18 |
| WH10009194 | A-II | 140 | 3755612 | 3756901 | 1290 | 1170 | 2 | Glyco_hydro_18 |
| WH10003963 | A-IV | 44 | 3647885 | 3649057 | 1173 | 1047 | 2 | Glyco_hydro_18 |
| WH10009310 | A-IV | 140 | 4097852 | 4099181 | 1330 | 1080 | 3 | Glyco_hydro_18 |
| WH10000184 | A-V | 195 | 712351 | 714343 | 1993 | 1137 | 3 | Glyco_hydro_18 |
| WH10004667 | A-V | 84 | 1696826 | 1698273 | 1448 | 1263 | 3 | Glyco_hydro_18 |
| WH10004720 | A-V | 84 | 1943371 | 1944863 | 1493 | 1440 | 1 | Glyco_hydro_18 |
| WH10008231 | A-V | 13 | 5347413 | 5351929 | 4517 | 1365 | 3 | Glyco_hydro_18 |
| WH10008749 | A-V | 140 | 2205113 | 2206420 | 1308 | 1191 | 2 | Glyco_hydro_18 |
| WH10006436 | B-I | 81 | 2786930 | 2787979 | 1050 | 1050 | 0 | GH18_chitinase_D-like |
| WH10000865 | B-I | 394 | 980828 | 981829 | 1002 | 1002 | 0 | GH18_hevamine_XipI_class_III |
| WH10002591 | B-I | 19 | 892234 | 893241 | 1008 | 954 | 1 | GH18_hevamine_XipI_class_III |
| WH10003851 | B-I | 44 | 2794213 | 2795364 | 1152 | 1095 | 1 | GH18_hevamine_XipI_class_III |
| WH10003001 | B-II | 19 | 3357251 | 3358748 | 1498 | 1173 | 3 | GH18_hevamine_XipI_class_III |
| WH10004309 | B-II | 84 | 628967 | 630185 | 1219 | 969 | 4 | GH18_hevamine_XipI_class_III |
| WH10002868 | B-V | 19 | 2531434 | 2532510 | 1077 | 1077 | 0 | GH18_CTS3_chitinase |
| WH10002282 | B-V | 16 | 3124941 | 3125942 | 1002 | 1002 | 0 | GH18_CTS3_chitinase |
| WH10003288 | C-I | 44 | 607411 | 611216 | 3806 | 3747 | 1 | GH18_chitolectin_chitotriosidase |
| WH10002109 | C-I | 16 | 2513862 | 2519348 | 5487 | 4221 | 14 | GH18_chitolectin_chitotriosidase |
| WH10009911 | C-I | 140 | 6014677 | 6019121 | 4445 | 3528 | 11 | GH18_chitolectin_chitotriosidase |
| WH10003350 | C-I | 44 | 930019 | 932820 | 2802 | 2661 | 2 | GH18_zymocin_alpha |
| WH10005062 | C-I | 84 | 3017609 | 3021222 | 3614 | 2565 | 8 | GH18_zymocin_alpha |
| WH10002350 | C-I | 16 | 3322645 | 3324428 | 1784 | 1638 | 2 | GH18_zymocin_alpha |
| WH10002829 | C-I | 19 | 2266811 | 2268792 | 1982 | 1617 | 6 | GH18_zymocin_alpha |
| WH10005656 | C-I | 84 | 5028566 | 5030541 | 1976 | 1596 | 6 | GH18_zymocin_alpha |
| WH10001817 | C-II | 16 | 825397 | 827224 | 1828 | 1431 | 5 | GH18_zymocin_alpha |
| WH10005213 | C-II | 84 | 3611927 | 3613581 | 1655 | 1362 | 5 | GH18_zymocin_alpha |
| WH10001816 | C-II | 16 | 824242 | 825375 | 1134 | 1134 | 0 | GH18_zymocin_alpha |
| WH10007469 | C-II | 13 | 2236163 | 2239948 | 3786 | 3564 | 4 | GH18_zymocin_alpha |
| WH10001630 | C-II | 257 | 2717848 | 2722337 | 4490 | 3891 | 8 | GH18_zymocin_alpha |
| WH10001666 | C-II | 16 | 287779 | 290059 | 2281 | 2040 | 2 | GH18_zymocin_alpha |
| WH10005780 | C-II | 81 | 355979 | 360265 | 4287 | 3546 | 5 | GH18_zymocin_alpha |
| WH10006445 | C-II | 81 | 2886438 | 2890875 | 4438 | 3732 | 5 | GH18_zymocin_alpha |
| WH10010026 | C-II | 140 | 6600623 | 6604947 | 4325 | 4113 | 3 | GH18_zymocin_alpha |
