# Supplementary material for: Genome-Wide Identification and Analysis of Chitinase GH18 Gene Family in Mycogone perniciosa
Source: Front Microbiol. 2021 Jan 11;11:596719. doi: 10.3389/fmicb.2020.596719 (PMC7829358; doi:10.3389/fmicb.2020.596719)
Supplement: Supplementary file 3 [file Table_3.docx]

**Table 3** Protein structure analysis of 41 GH18 genes of highly pathogenic strain Hp10 of *M. perniciosa*

| **Genes** | **Type** | **Length (aa)** | **MW (kDa)** | **PI** | **Instability index** | **Fat index** | **GRAVY** | **Localization predicted** |
| --- | --- | --- | --- | --- | --- | --- | --- | --- |
| WH10000025 | A-II | 342 | 37.13 | 6.32 | 28.45 | 73.98 | -0.193 | extracellular space |
| WH10000176 | A-II | 361 | 39.41 | 5.72 | 27.64 | 75.76 | -0.124 | extracellular space |
| WH10000259 | A-II | 362 | 39.44 | 6.09 | 29.9 | 74.75 | -0.173 | extracellular space |
| WH10003018 | A-II | 370 | 40.76 | 5.79 | 30.43 | 72.03 | -0.272 | organelle membrane |
| WH10003193 | A-II | 347 | 37.88 | 6.31 | 27.26 | 74.03 | -0.153 | extracellular space |
| WH10006636 | A-II | 362 | 39.56 | 5.9 | 30.79 | 72.32 | -0.184 | extracellular space |
| WH10006653 | A-II | 317 | 35.11 | 5.11 | 31.83 | 66.53 | -0.323 | extracellular space |
| WH10006907 | A-II | 305 | 33.28 | 5.86 | 31.87 | 73.31 | -0.241 | extracellular space |
| WH10009194 | A-II | 389 | 44.04 | 5.03 | 29.75 | 79.23 | -0.299 | extracellular space |
| WH10003963 | A-IV | 348 | 38.41 | 6.55 | 37.65 | 82.39 | -0.266 | Cytoplasm |
| WH10009310 | A-IV | 359 | 38.83 | 6.41 | 38.84 | 85.65 | -0.025 | extracellular space |
| WH10000184 | A-V | 378 | 41.81 | 4.78 | 29.66 | 74.39 | -0.396 | Cytoplasm |
| WH10004667 | A-V | 420 | 46.15 | 6.52 | 28.72 | 75.57 | -0.32 | extracellular space |
| WH10004720 | A-V | 479 | 52.31 | 5.23 | 37.22 | 73.9 | -0.365 | extracellular space |
| WH10008231 | A-V | 454 | 50.48 | 6.27 | 41.91 | 69.65 | -0.328 | Mitochondrion |
| WH10008749 | A-V | 396 | 44.40 | 4.6 | 35.81 | 69.04 | -0.564 | Cytoplasm |
| WH10006436 | B-I | 349 | 36.8 | 7.6 | 25.27 | 93.15 | 0.18 | extracellular space |
| WH10000865 | B-I | 333 | 35.32 | 5.06 | 31.75 | 75.38 | -0.122 | extracellular space |
| WH10002591 | B-I | 317 | 33.46 | 6.14 | 29.96 | 88.49 | -0.077 | extracellular space |
| WH10003851 | B-I | 364 | 39.93 | 4.89 | 39.68 | 65.96 | -0.321 | extracellular space |
| WH10003001 | B-II | 390 | 40.89 | 8.01 | 39.03 | 64.64 | -0.129 | extracellular space |
| WH10004309 | B-II | 322 | 34.65 | 6.23 | 26.6 | 77.61 | -0.025 | extracellular space |
| WH10002282 | B-V | 333 | 37.57 | 4.96 | 40.84 | 84.62 | -0.33 | Cytoplasm |
| WH10002868 | B-V | 358 | 39.87 | 6.13 | 37.26 | 84.75 | -0.156 | extracellular space |
| WH10003288 | C-I | 1248 | 135.25 | 5.42 | 41.39 | 64.79 | -0.411 | Mitochondrion |
| WH10002109 | C-I | 1406 | 150.78 | 4.86 | 42.24 | 71.34 | -0.135 | extracellular space |
| WH10009911 | C-I | 1175 | 130.16 | 6.76 | 32.1 | 81.5 | -0.216 | extracellular space |
| WH10003350 | C-I | 886 | 100.33 | 6.19 | 45.42 | 66.93 | -0.519 | Cytoplasm |
| WH10005062 | C-I | 854 | 96.48 | 6.68 | 33.94 | 63.1 | -0.564 | Cytoplasm |
| WH10002350 | C-I | 545 | 58.57 | 4.92 | 36.2 | 74.84 | -0.045 | plasma membrane |
| WH10002829 | C-I | 538 | 59.30 | 5.42 | 36.78 | 69.52 | -0.28 | endomembrane system |
| WH10005656 | C-I | 531 | 58.47 | 5.68 | 34.73 | 64.75 | -0.321 | plasma membrane |
| WH10001817 | C-II | 476 | 51.16 | 4.21 | 42.1 | 69.03 | -0.199 | extracellular space |
| WH10005213 | C-II | 453 | 50.71 | 4.66 | 41.42 | 70.02 | -0.518 | Cytoplasm |
| WH10007469 | C-II | 1187 | 124.89 | 5.99 | 48.14 | 73.84 | -0.003 | extracellular space |
| WH10001630 | C-II | 1296 | 138.06 | 6.11 | 34.28 | 75.69 | -0.089 | extracellular space |
| WH10001666 | C-II | 679 | 73.67 | 6.7 | 38.77 | 74.93 | -0.225 | extracellular space |
| WH10001816 | C-II | 377 | 39.96 | 4.44 | 36.06 | 72.52 | -0.158 | extracellular space |
| WH10005780 | C-II | 1181 | 128.06 | 5 | 31.36 | 73.29 | -0.32 | extracellular space |
| WH10006445 | C-II | 1243 | 135.6 | 5.7 | 33.38 | 76.28 | -0.288 | plasma membrane |
| WH10010026 | C-II | 1370 | 149.77 | 6.07 | 32.21 | 72.07 | -0.325 | extracellular space |
