# Supplementary material for: Genome-Wide Identification and Analysis of Chitinase GH18 Gene Family in Mycogone perniciosa
Source: Front Microbiol. 2021 Jan 11;11:596719. doi: 10.3389/fmicb.2020.596719 (PMC7829358; doi:10.3389/fmicb.2020.596719)
Supplement: Supplementary file 4 [file Table_4.docx]

**Table 4** The most significant DEGs in different infection stages

| **Infection stage** | **Protein ID** | **Putative function** | **P-value** | **Q-value** | **Expression patterns** |
| --- | --- | --- | --- | --- | --- |
| 3 vs 4 dpi | WH10001910 | Sugar (and other) transporter | 4.77E-10 | 8.29E-07 | upregulated |
| 4 vs 5 dpi | WH10006740 | AMP-binding enzyme | 3.62E-13 | 4.12E-12 | upregulated |
| 4 vs 5 dpi | WH10005671 | Ankyrin repeats (3 copies) | 1.83E-10 | 1.7E-09 | upregulated |
| 4 vs 5 dpi | WH10000037 | ANTH domain | 6.4E-16 | 8.76E-15 | upregulated |
| 4 vs 5 dpi | WH10009928 | Carbamoyl-phosphate synthase L chain, N-terminal domain | 2.47E-44 | 1.01E-42 | upregulated |
| 4 vs 5 dpi | WH10000866 | Carbohydrate-binding family 9 | 3.7E-61 | 2.94E-59 | upregulated |
| 4 vs 5 dpi | WH10000743 | CFEM domain | 5.68E-38 | 1.89E-36 | upregulated |
| 4 vs 5 dpi | WH10009972 | Cytochrome P450 | 1.09E-34 | 3.29E-33 | upregulated |
| 4 vs 5 dpi | WH10000403 | Fungal specific transcription factor domain | 0.0000817 | 0.0003945 | upregulated |
| 4 vs 5 dpi | WH10000342 | Glycosyl hydrolase family 92 | 3.38E-25 | 7.36E-24 | upregulated |
| 4 vs 5 dpi | WH10002227 | Heterokaryon incompatibility protein (HET) | 0.016321942 | 0.0474687 | upregulated |
| 4 vs 5 dpi | WH10009927 | LamB | 1.18E-25 | 2.61E-24 | upregulated |
| 4 vs 5 dpi | WH10006445 | LysM domain | 3.55E-40 | 1.24E-38 | upregulated |
| 4 vs 5 dpi | WH10001714 | Major Facilitator Superfamily | 0.004681443 | 0.015798 | upregulated |
| 4 vs 5 dpi | WH10002283 | Mechanosensitive ion channel | 1.33E-12 | 1.45E-11 | upregulated |
| 4 vs 5 dpi | WH10000836 | N-terminal domain of NWD NACHT-NTPase | 0.00000985 | 0.0000546 | upregulated |
| 4 vs 5 dpi | WH10006568 | Peptidase inhibitor I9 | 1.75E-171 | 8.07E-169 | upregulated |
| 4 vs 5 dpi | WH10005697 | Phosphate transporter family | 5.93E-51 | 3.23E-49 | upregulated |
| 4 vs 5 dpi | WH10008274 | short chain dehydrogenase | 3.56E-50 | 1.88E-48 | upregulated |
| 4 vs 5 dpi | WH10010023 | WSC domain | 0.000694871 | 0.0028338 | upregulated |
| 4 vs 5 dpi | WH10004197 | Zinc-binding dehydrogenase | 2.24E-48 | 1.09E-46 | upregulated |
| 5 vs 10 dpi | WH10008279 | Acetyltransferase (GNAT) domain | 5.46E-26 | 1.73E-24 | downregulated |
| 5 vs 10 dpi | WH10008187 | ANTH domain | 1.03E-51 | 1.14E-49 | downregulated |
| 5 vs 10 dpi | WH10003199 | ATPase family associated with various cellular activities (AAA) | 1.09E-18 | 2.18E-17 | downregulated |
| 5 vs 10 dpi | WH10003223 | Beta-ketoacyl synthase, N-terminal domain | 0.000168808 | 0.0006965 | downregulated |
| 5 vs 10 dpi | WH10008230 | Cyclin, N-terminal domain | 5.85E-45 | 4.86E-43 | downregulated |
| 5 vs 10 dpi | WH10003021 | Cytochrome P450 | 3.25E-104 | 1.45E-101 | downregulated |
| 5 vs 10 dpi | WH10005692 | FAD binding domain | 1.02E-85 | 2.87E-83 | downregulated |
| 5 vs 10 dpi | WH10000259 | Glycosyl hydrolases family 18 | 1.44E-30 | 6.41E-29 | downregulated |
| 5 vs 10 dpi | WH10000139 | Indoleamine 2,3-dioxygenase | 5.95E-19 | 1.22E-17 | downregulated |
| 5 vs 10 dpi | WH10006445 | LysM domain | 4.38E-65 | 6.93E-63 | downregulated |
| 5 vs 10 dpi | WH10003020 | Major Facilitator Superfamily | 8.58E-66 | 1.38E-63 | downregulated |
| 5 vs 10 dpi | WH10002881 | Meiotically upregulated gene family | 2.42E-18 | 4.71E-17 | downregulated |
| 5 vs 10 dpi | WH10002918 | Acyl transferase domain | 4.23E-35 | 2.36E-33 | upregulated |
| 5 vs 10 dpi | WH10001707 | Chitin synthase | 1.21E-184 | 1.18E-180 | upregulated |
| 5 vs 10 dpi | WH10002685 | Cytochrome P450 | 1.39E-31 | 6.57E-30 | upregulated |
| 5 vs 10 dpi | WH10003006 | Matrixin | 0.01505043 | 0.038037 | upregulated |
| 5 vs 10 dpi | WH10002449 | GppA phosphatase family | 0.001327168 | 0.0044838 | upregulated |
| 5 vs 10 dpi | WH10009603 | Ring finger domain | 3.97E-33 | 2.04E-31 | upregulated |
