# Supplementary figures and images for: Genome-Wide Identification and Analysis of Chitinase GH18 Gene Family in Mycogone perniciosa
Source: Front Microbiol. 2021 Jan 11;11:596719. doi: 10.3389/fmicb.2020.596719 (PMC7829358; doi:10.3389/fmicb.2020.596719)

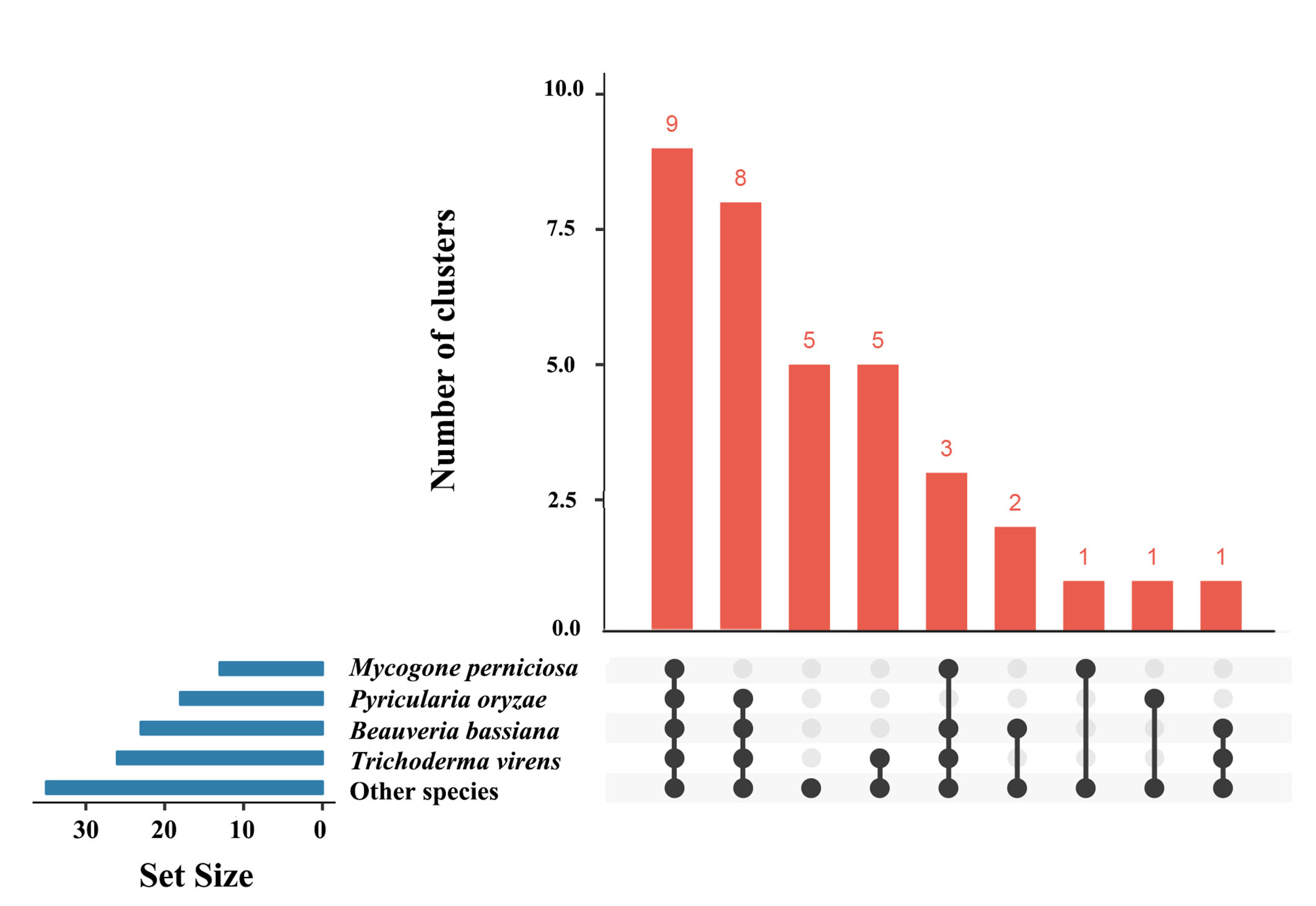

Supplement: Supplementary file 7 [file Image_1.jpg]
